# Benefits of Natural Antioxidants on Oral Health

**DOI:** 10.3390/antiox12061309

**Published:** 2023-06-20

**Authors:** Giuseppina Malcangi, Assunta Patano, Anna Maria Ciocia, Anna Netti, Fabio Viapiano, Irene Palumbo, Irma Trilli, Mariafrancesca Guglielmo, Alessio Danilo Inchingolo, Gianna Dipalma, Francesco Inchingolo, Elio Minetti, Angelo Michele Inchingolo

**Affiliations:** 1 Department of Interdisciplinary Medicine, University of Bari “Aldo Moro”, 70124 Bari, Italy; giuseppinamalcangi@libero.it (G.M.); assuntapatano@gmail.com (A.P.); anna.ciocia1@gmail.com (A.M.C.); annanetti@inwind.it (A.N.); viapianofabio96@gmail.com (F.V.); irenepalu@icloud.com (I.P.); irmatrilli@hotmail.com (I.T.); mariafrancescaguglielmo@hotmail.it (M.G.); ad.inchingolo@libero.it (A.D.I.); angeloinchingolo@gmail.com (A.M.I.); 2 Department of Biomedical, Surgical and Dental Science, University of Milan, 20122 Milan, Italy; elio.minetti@gmail.com

**Keywords:** natural antioxidant, oral health, oral disease, vitamins, ROS, reactive oxygen species, antioxidants, dental health, dentistry, oxidative stress

## Abstract

In recent years, special attention has been paid to the correlation between oxidation–reduction mechanisms and human health. The free radicals produced via physiological cellular biochemical processes are major contributors to oxidation phenomena. Their instability is the major cause of cellular damage. Free radical reactive oxygen species containing oxygen are the best-known ones. The body neutralises the harmful effects of free radicals via the production of endogenous antioxidants (superoxide dismutase, catalase, glutathione, and melatonin). The field of study of nutraucetics has found antioxidant capacity in substances such as vitamins A, B, C, E, coenzyme Q-10, selenium, flavonoids, lipoic acid, carotenoids, and lycopene contained in some foods. There are several areas of investigation that aim to research the interaction between reactive oxygen species, exogenous antioxidants, and the microbiota to promote increased protection via the peroxidation of macromolecules (proteins, and lipids) by maintaining a dynamic balance among the species that make up the microbiota. In this scoping review, we aim to map the scientific literature on oxidative stress related to the oral microbiota, and the use of natural antioxidants to counteract it, to assess the volume, nature, characteristics, and type of studies available to date, and to suggest the possible gaps that will emerge from the analysis.

## 1. Introduction

Oxidation–reduction reactions are physiologically produced during processes of aerobic cell metabolism and are indispensable for the very life of the organism. Free radical reacting oxygen species (ROS) containing oxygen are the best-known products of aerobic metabolism [1].

**Figure 1:**
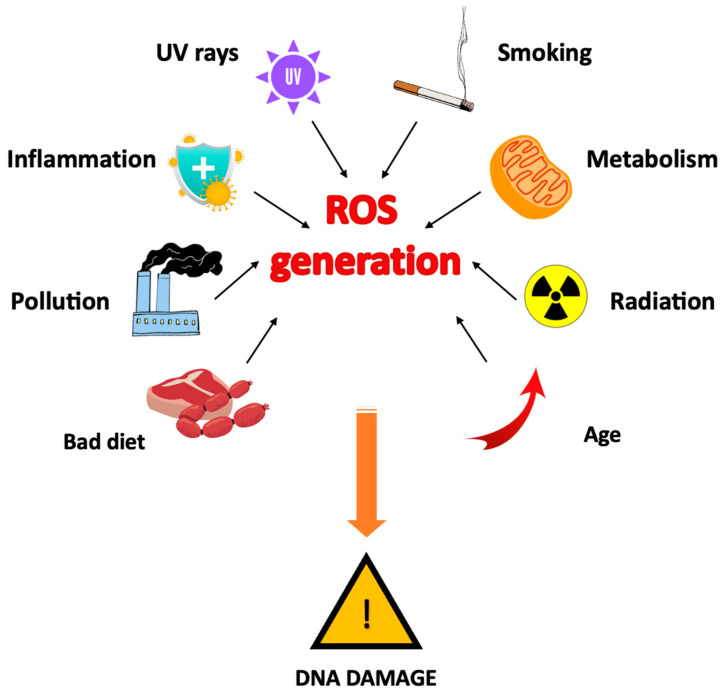
Factors inducing ROS formation.

Recent scientific studies increasingly confirm that the high presence of free radicals, resulting from the intensification of biochemical oxidation–reduction processes in cell metabolism, exerts a significant negative influence on both human health and healing ability. Free radicals, possessing unpaired electrons in their orbitals, have a propensity to form pairs, initiating chain reactions that lead to cellular damage [2,3]. The concentration of ROS increases as cell metabolism increases in response to hormonal and neuronal stimuli, and in pathological conditions (inflammation, ischaemia, diabetes, etc.) it increases further, and also increases in the presence of various environmental factors [4,5,6] (Figure 1).

The superoxide (the primary radical) originates when, through enzymatic and non-enzymatic reactions, an oxygen molecule receives an electron. Chain reactions can be triggered by the radical electron. The organism neutralises the harmful effects of free radicals with the production of endogenous antioxidants (superoxide dismutase, catalase, glutathione, and melatonin), thus protecting against oxidative damage from ROS, especially in the early stages [1,7].

To date, nutraceutics has identified several substances with antioxidant capacity, particularly polyphenols (PF), isoprenoids, vitamins A, B, C, D, and E, coenzyme Q-10, selenium, flavonoids, lipoic acid, carotenoids, and lycopene [8,9]. The control of free radical concentrations has fundamental importance not only to the balance of the gut microbiota but also to the oral microbiota (OMB) [10,11]. Most natural antioxidants can be administered through food. The Mediterranean diet (recognised as a UNESCO Intangible Cultural Heritage of Humanity), consisting of whole grains, pulses, extra virgin olive oil, fruit, vegetables and a small portion of animal protein, has been found to be the most appropriate one for adhering to correct diets and preventing the onset of diseases (cardiovascular diseases, neurodegenerative diseases and neoplasms) [12,13]. A lower oral cancer incidence rate correlates with higher fruit and vegetable consumption. Green tea (GT), curcumin (CUM), and resveratrol (RSV) are among the most-researched substances, containing large amounts of PFs, which have anti-inflammatory, antioxidant, neuroprotective, immunomodulatory, antitoxic, anti-apoptotic, anti-cancer and anti-diabetic properties. Recent research has shown that PFs also have epigenetic properties. PFs are able to influence the expression of genetic heritage, which is crucial in cancer therapy and its clinical course as well as in the potential control of the development of degenerative diseases [8,14,15].

GT and CUM, which are rich in natural antioxidant agents such as phenols, bioflavonoids, tetraterpenes, alkaloids and nitrogen, have been shown to inhibit the early stages and slow down the late stages of carcinogenesis in general and inflammatory processes [16].

### 1.1. Green Tea

GT extract, derived from a Southeast Asian plant, Camellia sinensis, has a significant role in the management of periodontal disease and caries [17]. In addition to containing protein, iron, magnesium, potassium, sodium, zinc, and vit. C, B1, B2, B3, and B6, it is rich in PFs known as catechins, including epigallocatechin-3-gallate (EGCG) and epigallocatechin (EGC) antioxidant substances capable of counteracting inflammatory processes and reducing the production of the main pro-inflammatory cytokines by the inflamed periodontal tissue (IL-1, IL-6, PGE2 and tumour necrosis factor alpha (TNF-alpha)), which are at the root of the onset of gingival infections and caries processes [18,19,20]. Catechin, especially EGCG, having antibacterial functions, stops the development and evolution of the disease. GT infusion, acting as a disinfectant, can be used as a mouthwash as well as for halitosis because it reduces the amount of volatile sulphur compounds produced by bacteria through their action on epithelial cells, salivary proteins and food residues. Administered after scaling and root smoothing sessions, it improves the healing process. Being rich in antioxidants, it protects against cell damage by inducing apoptosis in oral cancer cells, reducing the risk of oral cancer and slowing down its progression even in smoking patients [21]. GT has been recognised as having a chemopreventive and therapeutic function in oral cavity tumours, especially squamous cell tumours. It has been seen to modulate gene expression in oral tumours, reduce the activation of phase I enzymes arryl hydrocarbon hydroxylase (AHH), DT-diaphorase (DTD),cytochrome b 5, cytochrome P450, cytochrome b 5 reductase, cytochrome P450 reductase and increase phase II enzymes glutathione-S-transferase (GST) and UDP-glucuronyl transferase (UDP-GT) [22,23]. The difficulty in determining the onset of cancerous lesions from precancerous stages has been shown by the continuous administration of GT PFs. This could depend on changes in cell membranes, DNA/RNA/protein transcription systems from epigenetic factors, and the ability of tea PFs to influence cell activity [17]. GT by reducing the absorption of unsaturated fats, hyperlipidaemia, blood cholesterol concentrations and the onset of osteoporosis as well as the bacterial concentration in the oral cavity facilitates bone integration processes in dental implant surgery [24,25]. Despite its many beneficial effects, GT, containing caffeine, may cause anxiety, nervousness and insomnia if consumed in high quantities, as well as being hepatotoxic and affecting the functioning of the thyroid gland [26].

### 1.2. Curcumin

CUM, also known as diferuloylmethane, is the main polyphenolic substance found in the rhizomes of Curcuma longa. CUM is recognised as having pleiotropic functions (anti-inflammatory, antioxidant, neuroprotective, immunomodulatory, antitoxic, anti-apoptotic, anti-diabetic, anti-fertility, antimicrobial, anti-allergic, antidermatophytic, antidepressant, and cardioprotective ones) and even when administered in large quantities, it has no major side effects. Its important antioxidant activity (equal to that of vit. C and vit. E) is expressed in both water-soluble and fat-soluble solutions [8,27]. At the level of the oral cavity, it has been demonstrated that CUM can be an alternative disinfectant to oral disinfectants such as chlorhexidine, due to its ability to reduce the concentrations, biofilm formation and pathogenic effects of both S. mutans and Porphyromonas gingivalis in the short and long term (5 min–24 h), making it useful in preventing the onset of caries and periodontal processes. Studies have shown that treatment with CUM reduces gene expression related to the synthesis of extracellular polysaccharides and carbohydrates, and reduces the adhesion and transduction abilities of S. mutans and Porphyromonas gingivalis to the cell membrane [28,29].The latest research also suggests that CUM can reduce micronuclei and the colonisation of Candida albicans and Candida tropicalis, especially when combined with smoking cessation [30,31]. Since CUM is metabolised and eliminated quickly by the enzyme system in the bloodstream, its possibility, revealed through nanomedicine studies, to conjugate CUM to adjuvants and evaluate alternative delivery systems could be useful in prolonging half-life and bioavailability [32,33]. Some studies report some side effects, contraindications due to excessive CUM consumption, such as the stimulation of uterine contraction during pregnancy, reduced iron absorption and a reduction in both the number and mobility of spematozoa when administered orally, as well as the slowing down of coagulation. Therefore, in the case of planned surgery, it should be discontinued at least two weeks beforehand to avoid excessive bleeding [9,34].

### 1.3. Resveratrol

RSV (C 14 H 12 O 3: 3, 5, 4′-trihydroxy-trans-stilbene) is a phytoalexin and one of the best-known PFs used for its many therapeutic properties for human health. RSV is present in a plant called Polygonum cuspidatum, in the barks of some plants, in dried fruits (nuts and peanuts), flowers and red fruits (fermented grapes, mulberry, red wine, and blueberries). It is recognised in the isomeric forms trans-RSV and cis-RSV, the latter being more unstable [35]. RSV is recognised to have antioxidant, antimicrobial and anti-inflammatory, antitumour, antiviral, anti-ageing, antifungal and antithrombotic activities, as well as beneficial effects on diabetes, cardiovascular diseases, neurodegenerative diseases and specific bone metabolism [36,37]. Studies have shown that the administration of RSV reduces the onset of osteoporosis by increasing osteoblastic activity, reducing the risk of implant integration difficulties in prosthetic rehabilitation [38]. Furthermore, RSV blocks osteonecrosis of the jaws via the administration of bisphosphonates (especially zolendronate) and monoclonal drugs by combining it with platelet-containing growth factor concentrates (CGFs) [39,40,41,42,43,44,45]. At the bone level, RSV prevents the onset of Staphylococcus aureus osteomyelitis caused by Panton–Valentine’s leucocidin toxin (PVL) by blocking thrombotic events due to the damage produced to neutrophils [46,47].

RSV has antibacterial activity and prevents the biofilm formation of Porphyromonas gingivalis, as well as significantly reducing the virulence gene expression of the micro-organism. For these reasons, RSV could be considered a possible non-toxic and low-cost therapeutic choice for the treatment of periodontal disease [48]. PFs, especially those most widely used for their recognised properties (CUM and RSV), have a high degree of chemical–physical instability due to exposure to ultraviolet rays, high temperatures, alkaline and basic pH changes, poor water solubility, rapid deterioration in liver metabolism, and poor bioavailability in the oral cavity [49]. To overcome these limitations, strategies have been devised, realising, through nano-pharmaceuticals, drug delivery platforms or specific carriers capable of incorporating PFs, preserving their structural and therapeutic properties from deterioration and releasing them at the site of action [50]. This would allow the co-administration of PFs (lipophilic) with antibiotics (hydrophilic), such as Metronidazole and Ciprofloxacin, which are particularly used for the localised treatment of oral cavity diseases [51,52,53].

### 1.4. Vitamins

The role of vitamins, essential micronutrients, has been carefully evaluated in controlling human metabolic functions and also oral health [54]. Vitamin A, a fat-soluble vitamin, of both plant and animal origin, belongs to the carotenoid group (containing over 600 types), often found in yellow, orange and red vegetables, with powerful antioxidant activity, and controls epithelial integrity and the growth of teeth and bones [55]. It is used as a supplement in periodontal diseases, due to its reparative effects on epithelial cells. The recommended daily dose is at least a daily dose of 1000 IU (international units. Excessive doses of retinol, particularly β-carotene, can have teratogenic effects in pregnancy and hypopigmentation of the skin [56].

The B-vitamin group, containing B1 (thiamine), B2 (riboflavin), B3 (niacin), B5 (pantothenic acid), B6 (pyridoxine), B8 (biotin), B9 (folic acid), and B12 (cobalamin), with B1, B2, and B6–B12 being water-soluble, is of great importance in cell metabolism and tissue repair processes. B vitamins are contained in the liver, vegetables, egg yolk, and brewer’s yeast [57]. A lack of these vitamins leads to the disruption of the epithelial lining of the oral cavity and gingival bleeding. Some scientific studies note that the administration of vitamin B complex after periodontal surgery would ensure faster wound healing. In particular, vitamin B9 deficiency during pregnancy predisposes one to craniofacial malformations (cleft lip and palate) [58,59].

Vitamin C, or ascorbic acid, is water-soluble. It activates cell metabolism and the biosynthesis of amino acids, hormones and collagen and promotes iron absorption. Due to its strong antioxidant activity, it is an immunostimulant and prevents tumours, especially gastric cancer. Its administration ensures collagen synthesis and acts as an anti-ageing vitamin. Deficiency causes scurvy, with a clinical picture characterised by malaise, fatigue, recurrent inflammatory processes, gum bleeding, anaemia, lack of appetite, tooth loss, muscle pain and subcutaneous haemorrhages. Its administration is recommended in cases of oral mucosal lesions and after surgery [60].

Vitamin D is a fat-soluble vitamin. Two forms are recognised: cholecalciferol, produced by the body, and ergocalciferol, introduced through food. It is rarely found in food (some fatty fish, milk and dairy products, eggs, liver and green vegetables). It is found in large quantities in cod liver oil. Exposure to sunlight causes its synthesis in the body [61,62]. Its deficiency leads to rickets, bone deformities, osteoporosis and calcification defects of the teeth. Its administration, especially topically, is somewhat controversial, but it would seem that it can improve periodontal health due to an immunostimulating and anti-inflammatory action, as well as antibacterial by inhibiting the development and gene expression of the Porphyromonas gingivalis virulence factor [63]. Normal levels of vitamin D reduce the production of IL-8 and IL-6 at the periodontal level, reduce the virulence of Porphyromonas gingivalis, and stimulate the production of antimicrobial proteins (LL-37) in the gingival epithelium. By reducing osteoprotegerin synthesis and increasing Rankl action in osteoblasts, they activate alveolar bone metabolism with the activation and differentiation of osteoclasts promoting new bone remodelling [61,64,65,66]. Vitamin D deficiency would appear to be related to the increased likelihood of occurrence of osteonecrosis of the jaws due to bisphosphonates [67,68]. Vitamin D, due to its ability to inhibit cancer cell growth, has also been evaluated as a valid chemopreventive and therapeutic agent in head and neck squamous cell carcinomas [69].

The fat-soluble vitamin E, or tocopherol, has great antioxidant properties and is the most widely used of the vitamins favouring cellular activity. Its characteristics make it an important tool for cancer prevention—among other things, it protects the body against damage from pollution and cigarette smoke—as well as protein assimilation [70].

### 1.5. Aloe Vera

Aloe vera also plays a beneficial role in oral health due to its anti-inflammatory and soothing properties. Due to its antibacterial and anti-inflammatory properties, aloe vera can help fight infection and reduce inflammation of the gums. The application of aloe vera can provide relief from irritation, ulcers and mouth ulcers, accelerating the healing process of lesions, and can help maintain a healthy oral environment by counteracting the formation of bacterial plaque and contributing to periodontal health [71].

### 1.6. Propolis

Propolis also plays a significant role. Due to its antimicrobial, anti-inflammatory and antioxidant properties, propolis can help fight bacterial and fungal infections in the mouth. Useful in treating gingivitis and mouth ulcers, it reduces inflammation and speeds up the healing process. Propolis can also help prevent the formation of plaque and tartar, improving gum health and protecting teeth from cavities [72]. Due to a paucity of scientific publications on the therapeutic potential of nutraceuticals in the area of oral health and MB control, in relation to their use in nutrition, we have attempted to provide more detailed and in-depth information on the subject. Much still needs to be scientifically verified in relation to the constant provision of clinical evidence.

## 2. Materials and Methods

### 2.1. Search Processing

The PRISMA-ScR protocol was adhered to in conducting the present review [73]. A search was performed on the PubMed, Web of Science, Scopus, and Cochrane Library databases to identify articles relevant to the study topic, limited to those published in English from January 2018 to 30 April 30 2023. The search strategy utilised a combination of relevant terms to the study purpose, with the following Boolean keywords applied: “natural” AND “antioxidant”; “oral” AND “health”; “oral” AND “disease” (Table 1).

### 2.2. Inclusion Criteria

The relevant studies were assessed by the reviewers in pairs, based on the following inclusion criteria: (1) randomised controlled clinical trial design; (2) studies involvement of human participants of any age; (3) open-access publications; (4) studies written in the English language; and (5) studies investigating the positive effects of natural antioxidant compounds on oral health and oral diseases. Studies that failed to meet these criteria were excluded from consideration.

## 3. Results

**Figure 2:**
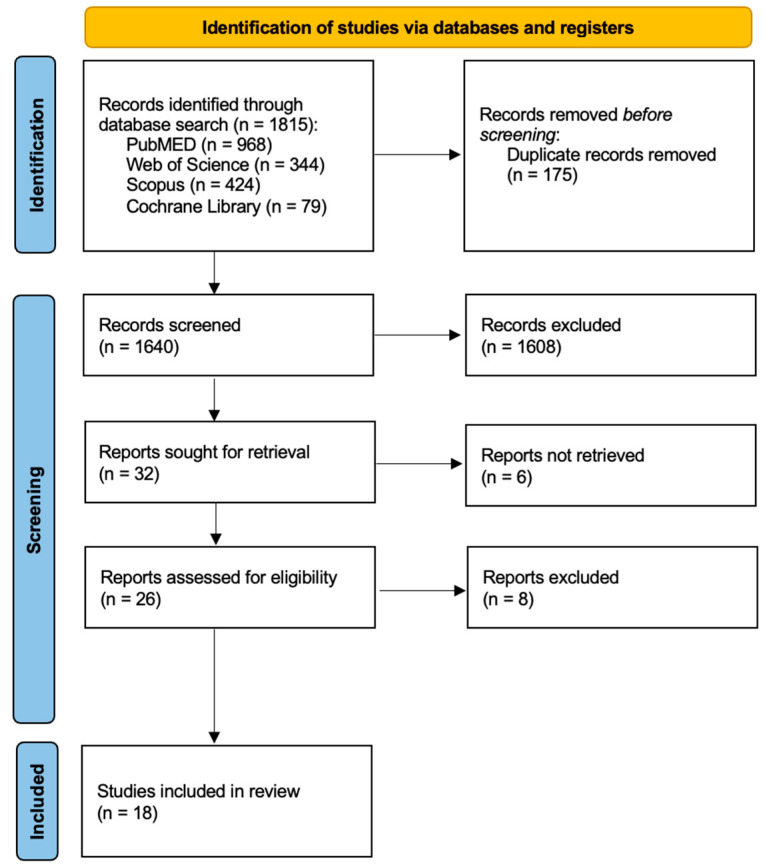
PRISMA-ScR Flow diagram.

A search across four databases, namely Pubmed (968), Web of Science (344), Scopus (424), and Cochrane Library (79), yielded a total of 1815 publications. After the removal of duplicates (175), 1640 records were screened based on the title and abstract, leading to the exclusion of 1608 articles. From the remaining 32 records, 14 papers were excluded after full-text evaluation, either for being off-topic or failing to meet the inclusion criteria. Ultimately, a total of 18 publications were deemed eligible for qualitative analysis (Table 2). The selection process is outlined in Figure 2.

## 4. Discussion

**Figure 3:**
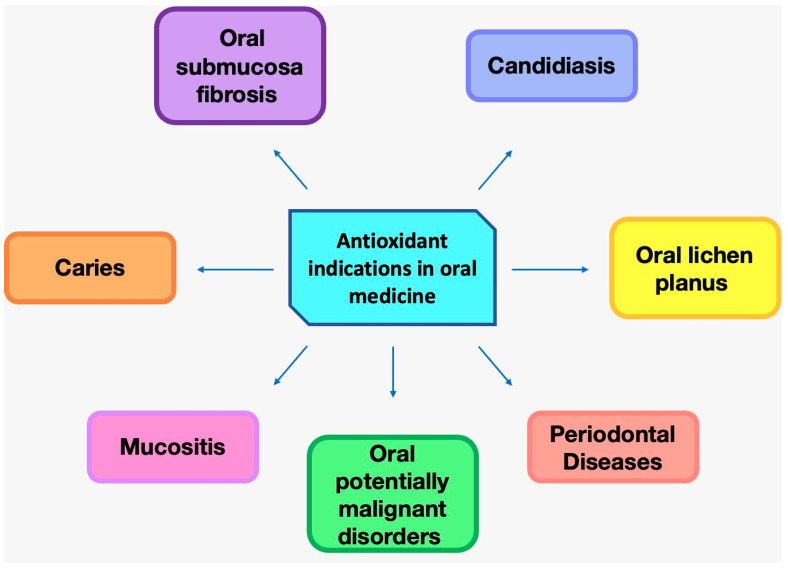
Oral diseases affected by antioxidant intake.

From the qualitative analysis of the articles included in the review, the oral diseases investigated for the evaluation of the potential beneficial effect of natural antioxidants are periodontal disease, mucositis, oral submucosal fibrosis, candidiasis, caries, oral lichen planus (OLP), and oral potentially malignant disorders (OPMDs) (Figure 3) (Table 3).

### 4.1. Periodontal Health

Periodontitis is a microbial challenge to the oral biofilm-induced immunoinflammatory host response that results in the breakdown of bone and connective tissue via biologically stimulated mechanisms. Pharmaceutical inhibition is recommended as an alternative strategy since pro-inflammatory mediators, arachidonic acid metabolites, and ROS are involved in pathogenesis [92].

In terms of nutritional intervention for host response modulation, it has been postulated that micronutrients including vitamins (C, E, A, and D), carotenoids, and PFs modulate pro-inflammatory cascades by acting as antioxidants to lessen oxidative stress [93].

**Figure 4:**
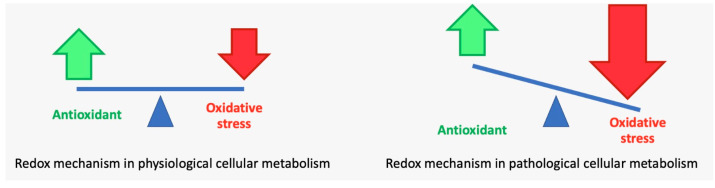
Redox mechanism in pathological and physiological cellular metabolism.

It has been demonstrated that vitamin C, a reducing substance that is water-soluble and donates electrons, maintains the equilibrium of cells’ redox potential and scavenges ROS generated by oxidative stress and the ensuing inflammatory responses [94] (Figure 4).

All cell membranes include the fat-soluble vitamin E, which prevents oxidative damage to membrane lipids [95].

It has been shown how both vitamins can help persons with gingivitis and periodontitis. Vitamin E might not have the same antioxidant effects as vitamin C because it is restricted to cell membranes and is not water-soluble. 

Synergistic effects may be anticipated when vitamin C is combined with other vitamins because it has been shown to reduce the vitamin E radicals created after scavenging oxygen radicals [96].

In the first four weeks of the test group’s comparison to the control group, Hong JY et al. [79] found a substantial improvement in the mean change of GI with a fixed-dose combination of vitamin C, vitamin E, lysozyme, and carbazochrome (CELC). The test group considerably decreased its GI at both 4 and 8 weeks after the baseline, whereas there was no appreciable difference in the control group. It is reasonable to assume that these identical outcomes are the consequence of SPR conducted equally for both groups given that mechanical plaque removal increases PI, PD, and CAL. Furthermore, even though the mean change was quite modest, it is still difficult to make a firm judgment regarding the therapeutic impact of CELC on gingival inflammation. To better understand its therapeutic efficacy, data from a larger sample size, full mouth examinations, and longer study periods should be gathered. 

Li X. et al. [81] examined the function of vitamin C in wound healing following the placement of dental implants both with and without bone grafts. The main finding of this clinical trial was that patients with CP who received GBR or Bio-Oss Collagen grafts had a greater capacity for healing after receiving postoperative vitamin C treatment. However, vitamin C supplementation did not reduce the discomfort experienced during dental implant surgery.

Instead, in their cross-sectional analysis, Li W. et al. [80] found a nonlinear relationship between the intake levels of a number of micronutrients, including vitamins B1 and E, and the prevalence of periodontitis. Their findings suggest that ingesting a variety of micronutrients, such as vitamin B1, within a specific range may help reduce the risk of developing periodontitis; however, consuming excessive levels of micronutrients might boost that risk (men should consume 90 mg of vitamin C daily and 1.8 mg of vitamin B1 daily). Their investigation also produced several results that differed from those of other studies. Earlier studies in the same population showed that vitamin C was a stimulator of periodontal health, but in this study, high vitamin C intake increased the likelihood of developing periodontitis [97,98]. The current study found that a variety of vitamin consumption deficiencies were associated with a higher prevalence of periodontitis when variables (such as gender, education, income, diabetes, alcohol use, etc.) were not taken into account. GSE, a naturally occurring polyphenolic compound, is found in the seeds of Vitis vinifera [99]. GSE is regarded as a powerful immunomodulator agent with anti-inflammatory, antioxidant, anti-microbial, and anti-carcinogenic properties [100]. The treatment of a variety of clinical disorders with microbial and inflammatory etiologies, particularly periodontal disease, depends on these physiologically advantageous features [101,102].

In patients with generalised periodontitis, Das M. et al. [82] proved the clinical effectiveness of the intra-pocket delivery of GSE as an adjuvant to SRP in the therapy of periodontal pockets. The results of the current experiment favour the use of GSE concentrate as an adjuvant to SRP in the mouth to improve clinical periodontal parameters. At the end of a three-month follow up, the current experiment demonstrated a substantial improvement in PI, GI, PD, and RAL for the test group compared to those of the control. Because the effect of GSE is dose- and concentrate-dependent, 4% GSE was used in this investigation for intra-pocket delivery. According to an in vitro investigation, GSE concentrations of between 0.5 and 2% are thought to have bacteriostatic effects, while concentrations of more than 2.5% are thought to have bactericidal effects [103].

Due to its improved antibacterial activity, the use of a high concentration of GSE in this trial compared to that used in the previous one may be responsible for the improvement in PD and RAL. Additionally, GSE’s cytoprotective effect on human gingival fibroblasts may help fibroblast proliferation resist oxidative stress brought on by periodontal inflammation. 

Sukmawati A.N. et al. [78] investigated the effects of curettage followed by 10% propolis subgingival irrigation on patients with CP. More than 300 active substances, including phenolic acids, flavonoids, terpenes, lipid wax compounds, beeswax, vitamins, proteins, amino acids, and sugar, are found in propolis. The majority of propolis’ biological actions are attributed to the quantity of flavonoids and caffeic acid phenethyl ester (CAPE) that it contains. Clinical measures (PI, PPD, and BOP) and immunological factors (IL-1 from gingival crevicular fluid) were assessed as part of the evaluation criteria. After 21 days, improvements were observed in all clinical measures and IL-1 concentration across all groups. Notable distinctions were found between groups A and B regarding the reduction in the PPD value (*p* < 0.05), reduction in the BOP value (*p* < 0.05), and reduction in the IL-1 concentration value.

The imbalance of the pro-oxidant and antioxidant system, which results in the accumulation of reactive oxygen species and toxic metabolites, especially diene and triene conjugates (DCs) and malonic dialdehyde (MDA) by lowering the level of antioxidant protection, is linked to the development of periodontitis [104]. Melnychuk et al. [76] investigated the potential for oxidase–antioxidant system indicators to be regulated in individuals with periodontitis under the influence of complicated therapy. As a result, and in line with the authors’ findings, it is advised to utilise an antioxidant and other bioregulators in complex periodontitis therapy. It was discovered that LP intensification and antioxidant defence system depletion increase when the dystrophic inflammatory processes in the periodontium become worse. 

Utilising the medication Spirulina platensis, which contains spirulina, a source of high-quality protein, vitamins, minerals, complex carbohydrates, vital amino acids, fats, and nucleic acids, it was feasible to achieve the clinical stabilisation of the disease [76]. 

Under the influence of a complex treatment, oxidase–antioxidant system indicators reduced in all patients throughout all follow-up periods (*p*2 < 0.05) and stayed relatively normal throughout the year (*p*1 > 0.05). Concurrently, CP activity increased and catalase activity and the iron saturation of TF decreased [105]. All patients saw an increase in TF catalase activity and iron saturation at all follow-up periods as a result of the therapy (*p* < 0.05; *p* < 0.05), and these results were only marginally different from those of healthy patients (*p* > 0.05). In the early grade and first grade, their full normalisation took six months to accomplish. In all groups, CP activity reduced and varied substantially from the baseline value after six months (*p* < 0.05) [76].

Gingivitis is an inflammatory process resulting from the interaction of a bacterial attack and host inflammatory response. With uric acid, albumin, ascorbic acid, glutathione, and other enzymes, saliva is the body’s natural antioxidant. Chemicals are released as part of the body’s defensive response against oral infections, inflaming the sensitive gingival tissues. Therefore, effective home therapies might be suggested to help orthodontic patients with gingivitis. The study’s objectives are to assess how well antioxidant essential toothpaste treats generalised chronic gingivitis in orthodontic patients and to suggest ways to improve the oral health of orthodontic patients. The study does not support the idea that using herbs as a treatment will be effective and safe [77].

Major pro-inflammatory cytokines produced by inflamed periodontal tissue include IL-1, IL-6, PGE2, and TNF-alpha, as well as ROS enzymes, proteins, host cells, ions, hormones, and markers of oxidative stress and antioxidants [106]. When produced during the respiratory burst, ROS are extremely toxic and destructive and are a key pathogenic mechanism for tissue damage and disease linked to phagocytic infiltration [107]. Antioxidant therapy serves as a preventative measure by helping to get rid of free radicals [108]. 

Lycopene and GT both have antioxidant properties that have been demonstrated to boost antioxidant status, lower inflammatory markers, and reduce oxidative stress, all of which are good for periodontal health [109,110].

According to Wasti et al., non-surgical treatment (scaling and root smoothing) and added antioxidant supplementation (lycopene + GT extract) have a direct favourable link to periodontal health. Notably, the test group that received antioxidant therapy showed statistically significant (*p* = 0.001) and higher improvements in clinical parameters than the control group did. The authors came to the conclusion that lycopene and GT extract oral supplements have a significant role in the management of periodontal disease and advised adopting an antioxidant-rich diet [74].

The effectiveness of melatonin as a dietary supplement used in conjunction with SRP for generalised chronic periodontitis (gCP) patients experiencing insomnia was examined by El-Sharkawy et al. In comparison to the placebo with SRP, the combination of melatonin and SRP enhanced the clinical attachment increase and decreased PD and salivary TNF levels. Additionally, oral melatonin reduced AIS scores in gCP insomniacs while improving sleep quality.

This could be explained by melatonin’s ability to act as a scavenger of exogenous and endogenous ROS and reactive nitrogen species (RNS) and, as a result, its ability to down-regulate pro-inflammatory cytokines released in periodontal tissues is related to its ability to act as an anti-inflammatory [111].

### 4.2. Mucositis

A common radiotherapy-induced side effect is mucositis of the oral cavity. In the literature, a standard treatment strategy for this inflammation has not yet been identified. Farzaneh Agha-Hosseini et al. in 2021 [87] conducted a randomised triple-blind clinical trial of 60 head and neck cancer patients undergoing radiotherapy treatment aged 18 and older with grade 3 or 4 mucositis according to WHO criteria. The patients were split into two teams: the study group and placebo group. In the placebo group, a combined mouthwash containing 0.2% vitamin E, which has an antioxidant effect, 0.1% triamcinolone as an anti-inflammatory agent and 0.2% hyaluronic acid, which protects tissue and increases cell proliferation, was administered for 4 weeks. The study found that patients with mucositis who were treated with this type of mouthwash experienced a reduction in inflammation and, consequently, a decrease in pain [87].

A similar study was conducted by Maede Salehi et al. [89] in 2018, a double-blind clinical trial which evaluated 50 patients 26–72 years with head and neck cancer undergoing chemotherapy with grade 3 or 4 mucositis according to WHO criteria. The patients were split into two 25-person teams. The first one was given two 50 mg tablets of Propolis each day for 21 days, whereas the second received a placebo. It was shown that the intervention group showed an important difference in the healing of oral mucositis. Therefore, the study concluded that Propolis is crucial for the treatment and prevention of chemotherapy-induced oral mucositis [89].

José Gonzalez-Serrano et al. [88] conducted a double-blind, randomised clinical trial in 2021 with the purpose of suggesting a treatment for PM. The treatment consisted of applying a gel with propolis extract, nanovitamin C and nanovitamin E three times a day for one month, all analysed through clinical and microbiological parameters. Two groups (the placebo group and intervention group) of 23 people each were identified. The study showed a complete resolution of PM of 26% in the intervention group in contrast to the control group with 0% resolution.

These studies indicate the benefits of antioxidants optimised PM and mucositis post chemotherapy/radiotherapy treatment [88].

### 4.3. Oral Submucosa Fibrosis

Ashwini Nerkar Rajbhoj et al. [90] conducted a randomised clinical trial for the management of oral submucosal fibrosis in 2021. The study’s objective was to assess the beneficial effects of CUM and aloe vera with subsequent oral mucosal physiotherapy. Sixty patients were divided into two groups of 30. In the first group, CUM gel was administered while in the other, aloe vera gel was administered. Both groups were also asked to perform oral physiotherapy exercises. At 2, 4 and 6 weeks, mouth opening and burning sensations were assessed. It was found that both types of gels improved symptomatology but that the aloe vera gel had a statistically significant result in resolving burning [90].

Muhammad Tahir et al. [91] in a prospective comparative study in 2021 compared the efficacy of alpha lipoic acid gel and aloe vera gel combined to that of intralesional steroids such as hydrocortisone, dividing 28 patients into two groups using the two different methods. Both were successful in reducing burning and opening of the mouth. It can therefore be deduced that alpha lipoic acid and aloe vera gel can be used as an alternative to steroids when the latter are contraindicated [91].

### 4.4. Candidiasis

Candida is a fungus commonly found in the oral cavity and can become pathological in the presence of changes in the oral microbiome, caused for example by the use of tobacco products [112]. Oral lesions caused by tobacco use can be detected via routine cytology under a light microscope, and colonisation by Candida can make lesions more susceptible to malignancy [113]. Therefore, it is important to monitor the oral health of individuals who use tobacco products and address any alterations in the oral microbiome or Candida colonisation that may increase the risk of oral cancer [114]. 

The purpose of the study by Mehta et al. [83] was to analyse the effect of CUM gel on the Candida oral colonies in subjects using tobacco products. Tobacco use is known to compromise oral hygiene, leading to alterations in the oral microbiome, including Candida. CUM, which has various properties, such as antioxidant, anti-inflammatory, antimicrobial, anticarcinogenic, antifungal and immunomodulatory ones, was selected for the study [83,115]. 

The intervention involved applying CUM gel to the lesion area 3 times a day for 2 months. The study involved 120 participants, divided into a study group (smokers) and a control group (healthy). All participants’ oral health was evaluated, followed by cytomorphometric analysis and Candida colonisation and speciation on CHROMagar [83]. 

The results revealed statistically significant variations in nuclear and cell diameter, and the number of micronuclei between the control and study groups. The Candida colonisation rate in the smoking group was substantially greater than that in the healthy group. Candida tropicalis was the most common in the research group, whereas Candida albicans was the most common in the control group [83]. A statistically significant change in nuclear diameter and quantity of micronuclei was detected in the smoking group following CUM administration. After CUM administration and smoking cessation, the number of Candida colonies decreased, and Candida albicans was the major species found in the research group. Overall, the results suggest that CUM was able to decrease micronuclei and the colonisation, together with smoking cessation [83].

### 4.5. Caries

Papaya (Carica papaya, from the Cariaceae family) is a plant native to the Moluccas Islands in Indonesia, which has spread to the tropical and subtropical areas of Central America, the Philippines, India and Africa [116]. The fruits, leaves, seeds, and latex of the plant have been used in folk medicine for a wide variety of medicinal purposes, from fungal infections and headaches to stomach problems. Although scientific evidence on the plant’s pharmacological efficacy is still lacking in many respects, its nutraceutical value is much more solid, translating into potential health benefits, especially due to the high presence of antioxidant vitamins and proteolytic enzymes, with proven antibacterial, antiviral and antifungal activity [117].

The pulp of the fruit contains valuable antioxidant nutrients: carotene; vitamins A, C, group B and E; flavonoids; minerals, including potassium and magnesium. The leaves and seeds of Carica papaya contain proteolytic enzymes (papain and chymopapain), useful for protein digestion; alkaloids (caroaine and caropasemine); polyphenolic compounds (benzyl isothiocyanates, tocopherols, β-carotene and carotenoids, and flavonoids); triterpenes [118].

The oil extracted from the seeds contains phenolic compounds, vanillic acid, vitamin C, oleic, palmitic, stearic, and linoleic acids.

The beneficial effects of this fruit in the oral cavity were investigated in the 2021 study by Cespedes et al. [84]. The aim of this study was to evaluate the antibacterial effect of Carica papaya mouthwash in inhibiting Streptococcus mutans. This was a double-blind RCT study performed on 40 participants divided into two groups: 20 subjects given Carica papaya mouthwash and 20 subjects given chlorhexidine. The antibacterial effect was verified by measuring the number of colony-forming units per millilitre (CFU/mL) of S. mutans in saliva samples taken at time 0, before rinsing with mouthwash, at time T1, 10 min after application, and at time T3, 8 days after mouthwash [84].

The results showed that the difference in CFU of S. mutans at time T0 and T1 with Carica papaya mouthwash was 764 CFU, while at time T3 the difference was 212 CFU. For the control group, which used 0.12% chlorhexidine, the number at time T0 and T1 was 683 CFU and at time T3 it was 455 CFU. However, when comparing the two groups using the t-test, no statistical differences were found between the CFU/mL of S. mutans at T0, T1 and T2 [84].

In conclusion, the study suggests that Carica papaya mouthwash could be a viable alternative for Streptococcus mutans inhibition, but further research is needed to confirm these results [84].

### 4.6. Oral Lichen Planus

OLP is a chronic inflammatory autoimmune disease that predominantly affects the oral mucosa in various clinical forms ranging from reticular to erythematous and ulcerative [119]. The frequency of disease is between 0.55 and 2%. The associated symptomatology is varied and may manifest as a burning sensation to severe pain [120]. Treatment aims to alleviate the symptoms and monitor the lesions in order to prevent any dysplastic evolution and includes corticosteroids administered topically or systemically. However, these drugs have several side effects; topically they cause a thinning of the mucosa, dysgeusia, stomatopirosis and secondary candidiasis; systemically they can cause osteoporosis, fluid retention, an increased susceptibility to infections and iatrogenic diabetes mellitus [120]. In order to avoid these side effects, researchers are continuously searching for alternative therapies for OLP; CUM, a natural phytochemical found in turmeric, has demonstrated therapeutic effects on several oral diseases due to its antioxidant, anti-inflammatory, antimicrobial, and anticarcinogenic properties. Previous studies have evaluated CUM’s effect on OLP, but these studies were limited by the drug’s poor bioavailability [121]. Kia et al. [85] analysed for the first time the therapeutic effects of oral nano-CUM in OLP; CUM mediates its anti-inflammatory effects by reducing transcription factors, enzymes and inflammatory cytokines and by inhibiting free radicals and nitric oxide.

The study compared the effectiveness of CUM and prednisolone in the management of OLP. Both groups, CUM and prednisolone, showed a decrease in the pain, burning sensation and lesions of OLP, with no significant difference between them [85]. Previous studies have shown that CUM can improve OLP symptoms and reduce pain severity, and some studies have shown that it has a higher recovery rate when used in combination with prednisone. However, the hydrophobic nature of CUM limits its bioavailability and solubility, which presents a challenge for its clinical translation into a practical therapeutic agent [85]. Nano-CUM, which has a higher dissolution rate, is being investigated as a potential solution. The study used nano-CUM in oral systemic capsules and found that a lower dose of 80 mg was effective at improving the symptoms of OLP compared to higher doses of non-nano-silicone forms [85]. The results suggest that the dose of CUM is more important than the duration of its use in improving OLP symptoms. However, studies with more follow up are needed to introduce nano-CUM as a new therapeutic agent in the management of OLP [85].

### 4.7. Oral Potentially Malignant Disorders

Oral cancer is often preceded by the occurrence of potentially malignant disorders (PMDs) such as leukoplakia, oral submucosal fibrosis (OSMF), and erosive OLP [122]. In 2–3% of cases, PMDs evolve into cancerous lesions, so chemoprevention is essential to invert, stabilise or stop their progression. An effective chemopreventive agent should be safe even after prolonged use, but retinoids, which are currently the most used drugs, have toxic side effects [123]. Among the natural dietary products most frequently analysed for their chemopreventive abilities, GT and CUM are of particular interest. These are readily available products with low toxicity and are capable of inhibiting carcinogenesis and reducing the risk of cancer [124]. In animal models, PFs such as EGCG present in GT and CUM demonstrated anticarcinogenic activity by inhibiting cell growth or the proliferation markers and regulating apoptotic cell death. Biomarkers such as p53, cyclin D1, and Ki67 can be used to value the efficacy of chemopreventive agents at the molecular level [125].

The study by Neetha et al. [86] examined the chemopreventive characteristics of GT and CUM on PMDs, as single agents or in combination. The combination treatment proved to be more effective and synergistic, due to its differentiated growth-inhibiting mechanism. GT and CUM are in fact rich in natural antioxidant agents such as phenols, bioflavonoids, tetraterpenes, alkaloids, and nitrogen, which have been shown to inhibit the early and late stages of carcinogenesis [86]. GT, rich in PFs, has been shown to inhibit various processes related to the development, evolution, and spread of cancer cells. When it is combined with CUM, the combination shows a synergistic action in inhibiting the growth of head and neck cancer and other tumour cell lines causing a good clinical response and a significant reduction in the biomarkers p53, cyclin D1 and Ki67 after three months of treatment. These results suggest that the combination of these natural antioxidant compounds may be an effective treatment for PMDs, with good safety and clinical response [86].

However, the treatment was ineffective at improving oral cancer-free survival. The results of this study indicate the efficacy of these agents in preventing the development of cancer or a second primary tumour [86].

## 5. Limits

A review of the literature shows that human models used in the study and evaluation of antioxidant principles in the oral cavity are scarcely used in the main studies present, so it is mandated and absolutely necessary that research can evolve towards an approach that does not only take animal models into account, but that can analyse the efficacy or otherwise of these compounds on large human subject populations.

## 6. Conclusions

In recent years, it has become more and more obvious how crucial it is to have an understanding of the benefits that nature provides so that new approaches and the tools can be used to combat the issues that medicine faces. Research is being conducted on natural chemicals that can improve human health through a number of biochemical interactions that contribute to the preservation of the equilibrium between antioxidants and ROS.

This is actually summarised in the term “ Nutraceutics”, a combination of the words “nutrition” and “pharmaceutics” which describes the branch of science that looks at all of the elements or active compounds in food that have a positive impact on health, illness prevention, and treatment. Antioxidants, whether organic or synthetic, can help to prevent many diseases early on and work best when they are present in high concentrations, but it is essential to recognise that, as things stand, further research with larger case studies and cross-sectional studies is needed to unravel and clarify the apparently conflicting results of some of the studies we have discussed. 

Furthermore, it is very important to emphasise how basic research cannot be separated from clinical/dental feasibility and how the study of the “microenviroment” within which these molecular mechanisms take place appears to be a road to be paved towards new discoveries.

## Figures and Tables

**Table 1:** Database search indicator.

Articles screening strategy	KEYWORDS: A: “natural”; B: “antioxidant”; C: “oral”; D: “health”; E: “disease”
	Boolean Indicators: (“A” AND “B”) AND ((“C” AND “D”) OR (“C” AND “E”))
	Timespan: from January 2018 up to 30 April 2023.
	Electronic Databases: PubMed, Web of Science, Scopus, and Cochrane Library

**Table 2:** Descriptive summary of item selection.

Author (Year)	Study Design	Number of Patients	Average Age (Years)	Materials and Methods	Pathology	Outcomes
Wasti et al., 2021 [74]	RCT	48 healthy patients (24 test group; 24 control group)	Unspecified	SRP and oral hygiene instructions. The test group was given a prescription for CLIK^®^, which contained GT extract (300 mg) and natural lycopene. Post-operative clinical parameter determination and uric acid estimation were performed 45 days later. Saliva was collected at baseline and the 45th day.	Gingivitis	Modified plaque index (PI) ↑,sulcular bleeding index ↑, and salivary uric acid levels ↑ in the test group. The test group’s gingival health improved more than the control group’s (improved antioxidant profile).
El-Sharkawy et al., 2019 [75]	RCT	74 patients with chronic periodontitis (CP) and primary insomnia (test group: 21 M, 17 F; control group: 20 M, 17 F)	Mean age: 45.6 in test group; 46.7 in control group	Patients in the test group received SRP and took 10 mg oral melatonin capsules once daily before bedtime for 2 months. The patients in the control group received SRP and took a placebo. The authors measured clinical attachment level (CAL) gain after 3 and 6 months of therapy.	Periodontitis	At 3 and 6 months, the melatonin group had ↓ probing depth (PD) and CAL compared to those of the placebo group. After 3 and 6 months, the melatonin group showed better PD↓ and CAL↑. No statistically significant differences in plaque index, gingival index, and bleeding upon probing (BOP%) between the two groups were found.
Melnychuk et al., 2022 [76]	Observational study	161 patients; 125 had periodontitis; 36 had intact periodontium	19–45	Exogenous and endogenous use of biologically active supplement based on blue-green microalgae Spirulina platensis, a paste made of spirulina powder and silica enterosorbent mixed with 0.05% solution of chlorhexidine bigluconate to a gel-like consistency, and Spirulina tablets were taken twice daily for four weeks.	Periodontitis	All patients had raised levels of malonic dialdehyde, diene conjugates, catalase activity, transferrin iron saturation, and ceruloplasmin activity, which were all reliably and long-lastingly regulated by the treatment. All indicators deviated marginally from the norm during the year, and periodontitis was stabilised.
Shasmitha et al., 2019 [77]	Clincial trial	42 orthodontic patients with generalised chronic gingivitis	14–30	Patients were assessed for gingivitis using GI, PD, blood on probing, and OHI. A modified Brass brushing technique using total care herbal toothpaste was demonstrated at the second session and the patients’ gingival condition was once more evaluated.	Gingivitis	The pre-treatment and post-treatment GI and OHI index scores were calculated with a 3-week time difference. Both scores were marginally higher than the respective pre-treatment GI and pre-OHI index scores, but neither was statistically significant.
Sukmawati AN et al., 2021 [78]	Comparative Study	6 patients	Unspecified	Group A received 10% propolis after curettage, while Group B received 1% tetracycline. PPD, BOP, and IL-1 concentration were measured at baseline on day 0, before curettage, and 21 days following curettage.	Periodontal disease	In patients with CP, 10% propolis as a subgingival irrigation agent improved the clinical parameters of periodontal tissue and concentration of IL-1 more effectively than 1% tetracycline did.
Hong JY et al., 2019 [79]	RCT	112 patients	19–80	Patients randomly received combinations of vitamin C and E, lysozyme, and carbazochrome (CELC) or the placebo for the first four weeks. Then, it the mean change in the GI was measured, followed by that in the PI, PD, and CAL at 4 and 8 weeks.	Periodontal disease	After 4 weeks and 8 weeks, the GI in the test group considerably ↓. When compared to a placebo, CELC significantly reduced gingival inflammation.
Li W et al., 2022 [80]	cross-sectional study	8959 patients	Mean age: 52.4	The US National Health and Nutrition Examination Survey (NHANES, 2009–2014) database was used to identify participants who underwent a periodontal examination and reported their micronutrient intake levels.	Periodontal disease	With adequate intake of the micronutrients (vitamin A, vitamin B1, vitamin B2, and vitamin E), the risk of periodontitis was decreased. A high consumption of vitamin B1 (1.8 mg/day for men and 1.3 mg/day for women), vitamin C (90 mg/day for men), and copper (1.1 mg/day combined) all raised the incidence of periodontitis.
Li X. et al., 2018 [81]	RCT	128 patients	Mean age: 44.9	Group A received dental implants supported by the guided bone regeneration (GBR) technique; group B received dental implants with Bio-Oss Collagen; group C received dental implants, containing patients with CP; and group D received dental implants without any bone grafting or periodontal disease. Each group was split into a control subgroup and an experimental subgroup that received vitamin C.	Periodontal disease	Supplementing with vitamin C helps patients with CP and those receiving GBR or Bio-Oss Collagen grafts heal more quickly after receiving dental implants.
Das M. et al., 2021 [82]	RCT	72 patients	Mean age: 39.2 ± 8.6	Randomly, two groups of patients with periodontal pockets were formed; the test group received an intra-pocket administration of grape seed extract (GSE) along with SRP, and the control group received SRP alone. At baseline and three months, clinical measurements including the PI, GI, PPD, and relative attachment level (RAL) were taken.	Periodontal disease	At the end of 3 months, the test group showed PD↓ and RAL↓; there was no discernible difference between PI and GI. It might be advantageous to control periodontal pockets via the intra-pocket application of GSE and SRP.
Mehta et al., (2022) [83]	Prospective study	120 (60 cases: 36 M 24 F-60 controls:31 M 29 F)	18–40	CUM gel was applied 3 times a day for 2 months; cytomorphometric analysis using CHROMagar was performed	Candidiasis in tobacco users	CUM reduces both the number of micronuclei and also Candida colonies.
Cespedes et al., (2021) [84]	Double-blinded RCT	40 (20 cases-20 controls)	Unspecified	Comparison between Carica papaya and chlorhexidine mouthwash	Caries	Carica papaya has anti S. mutans action which is comparable to that of chlorhexidine
Kia et al., (2020) [85]	Double-blinded RCT	57 (29 case-28 controls)	51,86 (CUM-cases) 53.67 (prednisolone-controls)	Comparison between administration of 80 mg CUM in the Nano-Micellar Soft gel capsule and 10 mg Prednisolone in capsules	Oral lichen planus (OLP)	CUM may represent an alternative therapy in patients in which corticosteroids are contraindicated; it is effective in preventing the recurrence of OLP lesions.
Neetha (2020) [86]	Double-blinded Randomised preliminary study	60 (20 GT; 20 CUM; 20 combination)	45.5 (16–82)	Topical + systemic administration for 3 months of GT extract (800 mg/day), CUM (950 mg/day) or both combined. Evaluation of biomarkers Ki67, cyclin D1 and p53 at time 0 and at 12 weeks was performed	Oral potentially malignant disorders (OPMDs)	The combination of CUM and GT extract has synergistic action, causing clinical benefits and the downregulation of molecular biomarkers after 12 weeks.
Farzaneh Agha-Hosseini et al,. 2021 [87]	RCT	60 patients	least 18 years old (no maximum age limit)	Combined mouthwash was prepared with 0.1% triamcinolone, 0.2% vitamin E and 0.2% hyaluronic acid	Oral mucositis caused by radiotherapy	The reduction in inflammation and pain was significantly higher in the intervention group.
José Gonzalez-Serrano et al., 2021 [88]	double-blind RCT	46 patients	around 60 years old	Gel containing propolis extract, nanovitamin C and nanovitamin E	Oeri-implant mucositis (PM)	Full PM resolution was observed in the intervention group.
Maede Salehi et al., 2018 [89]	double-blind clinical trial	50 patients	26–70	2 tablets of 50 mg propolis daily	Oral mucositis caused by chemotherapy	The intervention group achieved a significant difference in the healing of oral mucositis.
Ashwini Nerkar Rajbhoj et al., 2021 [90]	RCT	60 patients	15–55	CUM gel and aloe vera gel with oral physiotherapy	Oral submucous fibrosis (OSF)	Both types of gel improved the symptoms but aloe vera gel achieved a statistically significant result in remedying burning.
Maometto Tahir et al., 2021 [91]	Prospective comparative study	28 patients	Mean age: 26.14 ± 5.33	Comparison between alpha lipoic acid with aloe vera gel and hydrocortisone	OSF	Comparison between the alpha lipoic acid with aloe vera gel group versus the hydrocortisone group showed almost similar results.

**Table 3:** Schematic summary of the examined factors.

Oral Disease	Nutraceutical/Food Substance
***Periodontal disease***	Propolis, vitamin C, vitamin E, vitamin A, vitamin B2, copper, sage essential oil, grape seed, green tea, uric acid, menthol and thymol essential oils, ferulic acid and phloretin, and melatonin
***Mucositis***	Vitamin E, triamcinolone and hyaluronic acid, propolis, and vitamin C
***Oral submucosal fibrosis***	Curcumin and aloe vera
***Oral candidiasis***	Curcumin
***Caries***	Papaya
***Lichen planus***	Curcumin
***Malignant oral cavity disorders***	Green tea and curcumin

## Data Availability

Not applicable.

## Abbreviations

ROS	Reactive Oxygen Species
GT	Green tea
CUM	Curcumin
RSV	Resveratrol
PFs	Polyphenols
EGCG	Epigallocatechin-3-gallate
EGC	Epigallocatechin
TNF-alpha	Tumour necrosis factor alpha
AHH	Arryl hydrocarbon hydroxylase
DTD	DT-diaphorase
GST	glutathione-S-transferase
UDP-GT	UDP-glucuronyl transferase
CGFs	Platelet-containing growth factor concentrates
PVL	Panton–Valentine’s leucocidin
IU	International units
SBI	Sulcus bleeding index
PD	Probing depth
CAL	Clinical attachment level
PI	Plaque index
GI	Gingival index
BOP	Bleeding on probing
OHI	Oral hygiene index
PPD	Probing pocked depth
IL-1	Interleukin 1
CELC	Vitamin C, vitamin E, lysozyme, and carbazochrome
GBR	Guided bone regeneration
GSE	Grape seed extract
SRP	Scaling and root planing
RAL	Relative attachment level
OLP	Oral lichen planus
OPMDs	Oral potentially malignant disorders
OSF	Oral submucous fibrosis
CAPE	Caffeic-acid-phenethyl-ester
DCs	Diene conjugates
MDA	Malonic dialdehyde
CP	Chronic periodontitis
IL-6	Interleukin 6
PGE2	Prostalgandin E2
gCP	Generalised chronic periodontitis
WHO	World Health Organization
PM	Peri-implant mucositis
CFU/mL	Colony-forming units per millilitre
